# The Application of Mask Region-Based Convolutional Neural Networks in the Detection of Nasal Septal Deviation Using Cone Beam Computed Tomography Images: Proof-of-Concept Study

**DOI:** 10.2196/57335

**Published:** 2024-09-03

**Authors:** Shishir Shetty, Auwalu Saleh Mubarak, Leena R David, Mhd Omar Al Jouhari, Wael Talaat, Natheer Al-Rawi, Sausan AlKawas, Sunaina Shetty, Dilber Uzun Ozsahin

**Affiliations:** 1 Department of Oral and Craniofacial Health Sciences College of Dental Medicine University of Sharjah Sharjah United Arab Emirates; 2 Operational Research Center in Healthcare Near East University Nicosia Turkey; 3 Department of Medical Diagnostic Imaging College of Health Sciences University of Sharjah Sharjah United Arab Emirates; 4 Department of Preventive and Restorative Dentistry College of Dental Medicine University of Sharjah Sharjah United Arab Emirates

**Keywords:** convolutional neural networks, nasal septal deviation, cone beam computed tomography, tomographic, tomography, nasal, nose, face, facial, image, images, imagery, artificial intelligence, CNN, neural network, neural networks, ResNet

## Abstract

**Background:**

Artificial intelligence (AI) models are being increasingly studied for the detection of variations and pathologies in different imaging modalities. Nasal septal deviation (NSD) is an important anatomical structure with clinical implications. However, AI-based radiographic detection of NSD has not yet been studied.

**Objective:**

This research aimed to develop and evaluate a real-time model that can detect probable NSD using cone beam computed tomography (CBCT) images.

**Methods:**

Coronal section images were obtained from 204 full-volume CBCT scans. The scans were classified as normal and deviated by 2 maxillofacial radiologists. The images were then used to train and test the AI model. Mask region-based convolutional neural networks (Mask R-CNNs) comprising 3 different backbones—ResNet50, ResNet101, and MobileNet—were used to detect deviated nasal septum in 204 CBCT images. To further improve the detection, an image preprocessing technique (contrast enhancement [CEH]) was added.

**Results:**

The best-performing model—CEH-ResNet101—achieved a mean average precision of 0.911, with an area under the curve of 0.921.

**Conclusions:**

The performance of the model shows that the model is capable of detecting nasal septal deviation. Future research in this field should focus on additional preprocessing of images and detection of NSD based on multiple planes using 3D images.

## Introduction

### Background

Nasal septal deviation (NSD) is characterized by malalignment of the nasal septum with reference to the anatomical midline [1,2]. NSD has been associated with turbinate hypertrophy, decreased air volume, and diseases of the nasal cavity and maxillary sinus [3-5]. The prevalence rates of NSD vary from 26% to 97%, as reported in studies originating from different parts of the world [3-6].

The contemporary diagnostic modalities used for the evaluation of NSD include anterior rhinoscopy, endoscopy, computed tomography, and magnetic resonance imaging [7-9]. Over the past few years, cone beam computed tomography (CBCT) has established itself as a low radiation diagnostic modality for imaging of nasal and paranasal sinus [10-12].

In conventional radiology practice, radiologists analyze images to detect and monitor disease conditions. However, recent advancements in the field of artificial intelligence (AI) technologies have enabled the automatic recognition of intricate patterns of imaging data. Recent studies have revealed that the accuracy and reproducibility of AI-based radiology evaluations aid radiologists in image interpretation and diagnosis [13].

A recent scoping review revealed a growing number of recent research articles on the application of AI for detecting anatomical nasal and paranasal landmarks using various imaging modalities [14]. Recently published studies have revealed that deep learning models are effective in detecting various conditions, ranging from nasopharyngeal carcinoma to nasal bone fractures across different imaging modalities [15-17]. To the best of our knowledge, no studies have used AI models to detect NSD in CBCT image slices. This AI model could serve as an adjunct to physicians in the radiographic detection of NSD.

### Aim of This Study

The aim of this research was to develop a real-time AI model to detect NSD and determine its accuracy in detecting NSD in CBCT images.

## Methods

### Data Source

We collected 204 coronal CBCT images of the nasal septum (138 with a deviated nasal septum and 66 with a nondeviated nasal septum) from the dental radiology archives of University Dental Hospital, Sharjah. The CBCT scans were obtained using a Planmeca Viso 7 CBCT unit (0.2 mm resolution, 95 kVp, and 5mA; Finland) and large fields of view (FOVs) from patients who visited the dental hospital between June 1, 2020, and June 30, 2023. CBCT scans of male and female patients within the age group of 18 and 60 years were included in the study. CBCT scans with small and medium FOV, incomplete scans, and artifacts were excluded. Scans of patients with a history of midfacial trauma, surgery, cleft palate, and complete nasal obliteration were also excluded.

### Ethical Considerations

This study was reviewed and approved by the Research Ethics Committee of the University of Sharjah (REC 21-01-10-01), which waived the requirement for patient consent. Only preexisting CBCT scans in the radiology archives of the dental teaching hospital were used for the study. No new CBCT scans were done for the study. No personal identification details of the patients were used during the analysis of CBCT scans in the study. No compensation was provided to the participants whose CBCT scans were used in the study.

### Procedure

**Figure 1:**
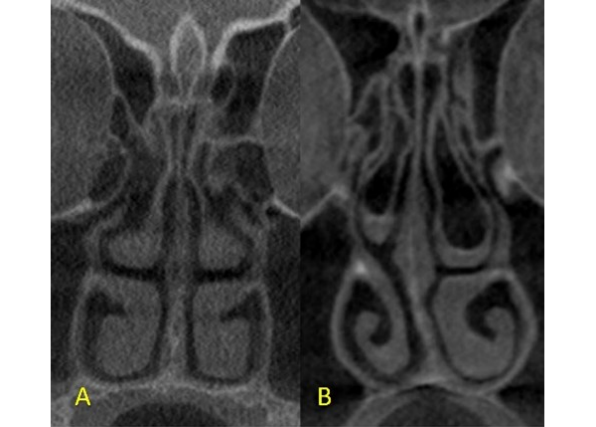
Cropped coronal cone beam computed tomography images showing (A) a nondeviated nasal septum and (B) a deviated nasal septal deviation.

The assessment of CBCT was performed directly on a 1920 × 1080–pixel and 23-inch DELL monitor screen. Since there were only 210 CBCT scans with a large FOV in the radiology archive, convenience sampling was used in the study, with only 204 scans. A total of 6 scans were not used due to meeting 1 or more exclusion criteria. Of the 204 CBCT images, 163 were assigned to the training group, and the remaining 41 of the CBCT images were assigned to the testing group on a random basis using a computerized random number generator. Uniformity was maintained while cropping the coronal image fields in all the CBCT scans so that the anatomical landmarks were consistent. Each image was then cropped into a 200 × 400–pixel square, extending from the crista galli superiorly to the hard palate inferiorly, and 5 mm laterally from the lateral nasal wall on both sides (Figure 1A and 1B). The files were saved in JPEG format.

**Figure 2:**
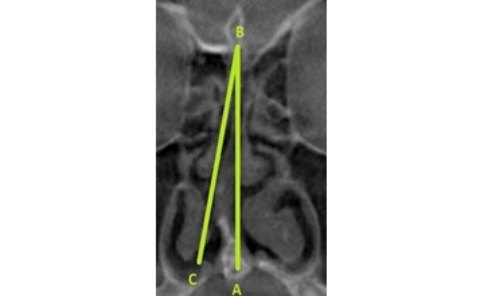
Point A represents the junction of the nasal septum with the floor of the nasal cavity. Point B represents the Crista Galli. The line BC represents a tangent drawn from point B and passing through the outermost part on the convexity of the deviated septum.

Two maxillofacial radiologists classified the nasal septum images into normal or deviated. In case there was a disagreement, a third radiologist was consulted for the finalization of the classification. NSD was determined by the method used by Shetty et al [11] and Al-Rawi et al [18] (Figure 2).

**Figure 3:**
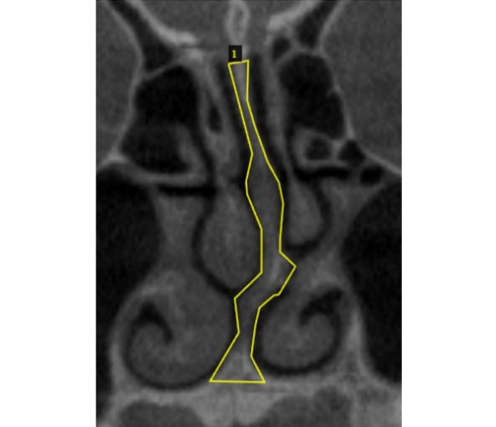
An image annotated using the Visual Geometry Group Image Annotator software showing a deviated nasal septum.

**Figure 4:**
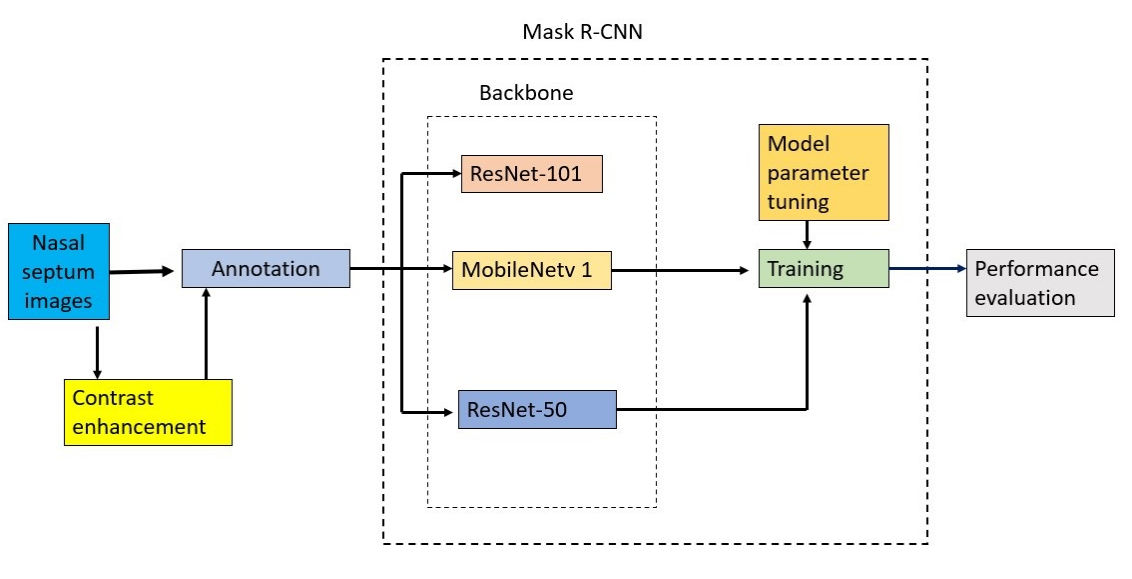
The flowchart of the study describing the stages of contrast enhancement, annotation, model training and performance evaluation. RCCN: region-based convolutional neural networks.

**Figure 5:**
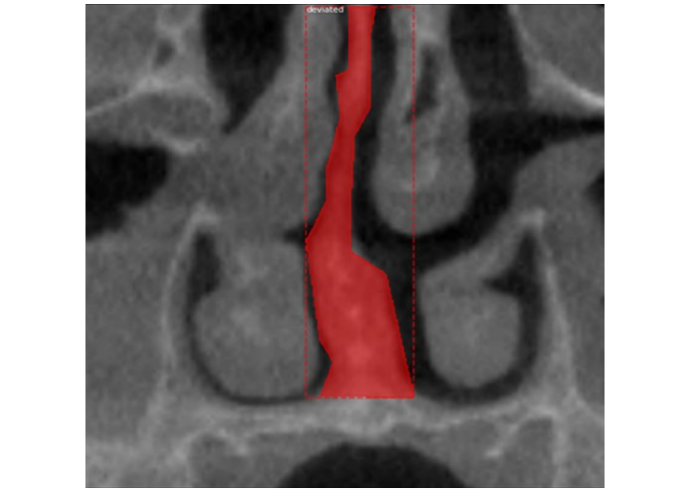
Detection of nasal septal deviation in the cropped coronal cone beam computed tomography image by Mask region-based convolutional neural networks.

**Figure 6:**
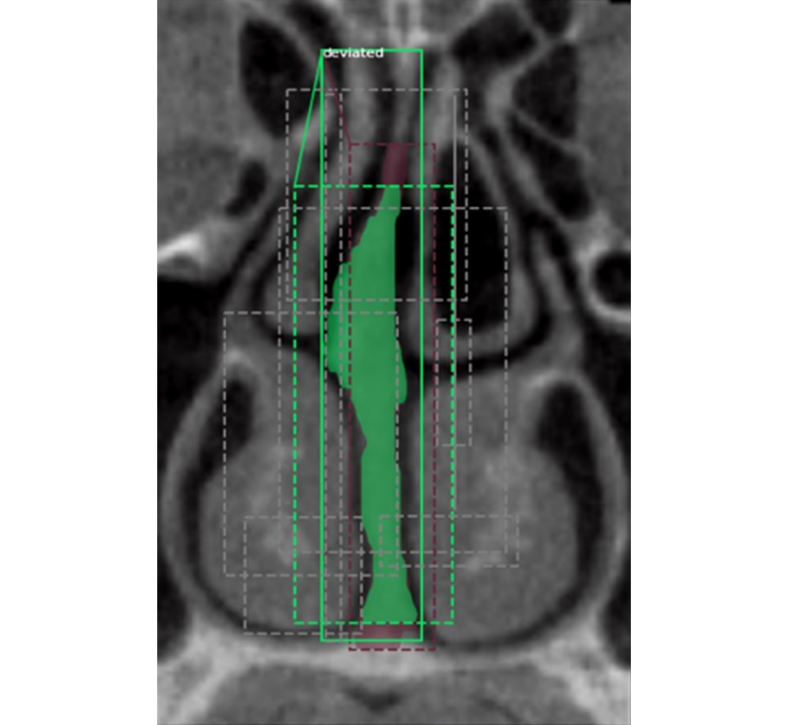
Region proposals in the cropped cone beam computed tomography image showing potential areas of interest (10 random Regions of Interest out of 200).

The annotations were done using the Visual Geometry Group Image Annotator manual annotation open-source software (Figure 3) [19]. After annotation, the data (cropped coronal images) were used for training (80%) and testing (20%) of the AI model. In training the models, we only considered images with a deviated septum and discarded images with a nondeviated septum. We also augmented the training data to increase the data to 5 times the original number of the deviated images. The process of NSD detection using Mask region-based convolutional neural networks (R-CNN) is described in Figure 4. Figures 5 and 6 show the test image with detection results and examples of region proposals, respectively.

### Mask R-CNN

To identify targets and detect deviated nasal septum, Mask R-CNN was used to segment the nasal septum at the pixel level. The Mask R-CNN architecture consisted of faster R-CNN, Region of Interest (RoI) alignment (RoIAlign) technique, and feature pyramid networks [20]. Mask R-CNN is an improved version of faster R-CNN, a model used for object detection, which includes a segment prediction branch for each RoI, allowing it to perform both detection and pixel-level segmentation at the same time. Mask R-CNN’s dual nature allows it to serve as both an object identification model and an excellent tool for instance segmentation tasks [21-23].

### The Model Training

Mask R-CNN with a pair of different backbones—ResNet50, ResNet101, and MobileNetV1—was used in this work to detect and segment deviated nasal septum, and weights from trained models were adopted using the transfer learning approach to assess the performance of the proposed detection model. The models were developed for 2 distinct scenarios. In the first instance, the model was developed using the original images and the ResNet50, ResNet101, and MobileNetV1 backbones. In the next instance, the model was trained with contrast enhancement (CEH) along with the aforementioned backbones. The performance of the models based on the mean average precision (mAP) and area under the curve (AUC) was compared to find the best model. To boost the total number of images and the model performance, the dataset was augmented before the model was trained by randomly flipping, scaling, and rotating the images along the x-axis and the y-axis. This increased the original training data by 5 times. Additionally, augmenting the data stops the model from becoming overfit. The hyperparameter values for the model were set as follows: weight decay=0.0001, learning rate=0.001, detection minimum confidence=0.9, learning momentum=0.9, mask pool size=14, validation steps=100, steps per epoch=100, and epoch=50.

mAP is widely used in object detection models because it efficiently integrates recall and precision into a single, all-encompassing statistic, which is essential for assessing localization efficacy and detection accuracy. Its ability to determine the average precision for every class independently is important since it provides a thorough understanding of the model’s performance in a variety of object classes. Crucial to object detection tasks, mAP includes varying intersection over union thresholds to evaluate the model’s accuracy in object localization. This metric has been standardized in well-known benchmarks, such as MS COCO [24] and PASCAL VOC [25]. As a result, it is a widely recognized tool for comparing performance between various models and datasets and for producing a single, all-inclusive figure that sums up the overall effectiveness of object detection models. mAP and AUC detection levels were used to evaluate model performances. The training environment used was Anaconda and Jupyter notebook, Keras 2.0.8, and tensor flow 1.4.0 on a GeForce Nvidia GTX1080, with an i7 processor and a RAM of 16 GB.

### MobileNetV1

A network model called MobileNet uses depth-dependent separable convolution as its basic component [26]. It has 2 levels, called convolution layers, independently for depthwise and point convolutions. The output feature maps of the preceding convolution layer are superimposed on the input feature maps of each dense block layer. There is a transition layer in DenseNet between 2 dense blocks. The quantity of input feature maps is reduced in the transition layer using a 1 × 1 convolution kernel. In place of a pooling layer, MobileNet depends on a convolution layer because it lacks a transition layer. The convolution layer automatically convolves the output feature map of the previous point convolution layer with stride 2, thereby decreasing the overall dimension of the feature map [26].

### ResNet

To solve a problem in computer vision, machine learning professionals use deep convolutional neural networks in combination with additional layers. A deeper network could promote the degrading problem even though the number of stacked layers may improve the model’s properties. Since discrete layers may be trained for various jobs to generate highly accurate results, these additional layers help in the quicker convergence of complex problems. This drop in performance was not brought on by overfitting. The network configuration, the optimization technique, and the problem with vanishing gradients may be to blame. Deep residual networks use residual blocks to improve the models’ performance. By establishing a different path for the gradient to take, they can address the issue of vanishing gradients. They also allow the model to learn an identity function, which guarantees that the model’s top layers perform on comparable levels with its bottom layers. ResNet was created specially to deal with this problem [27,28].

The ResNet50 architecture is based on the ResNet34 paradigm, except that every component is composed of a series of 3 layers instead of 2. This model is far better than the 34-layer version of ResNet and produces 3.8 billion floating-point operations per second. Each of the preceding 2-layer blocks was swapped out for a 3-layer bottleneck block to produce a 50-layer design [29].

### Performance Evaluation

Precision is an important metric to assess a model’s performance in object detection. It evaluates the precision of the objects detected, emphasizing the percentage of accurately predicted objects that are relevant or true. With an emphasis on the context of detected objects, the formula for precision in object detection is comparable to that of other classification tasks, as follows:



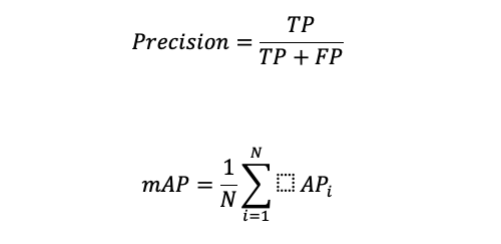



where precision (P) is the ratio of true positives (TPs) to the sum of TPs and false positives (FPs), and N is the number of instances.

### Contrast Enhancement

To improve a picture’s visual quality, CEH increases the contrast between different image areas. It aims to increase the variety in pixel intensity values to enhance the visual appeal of the image and the discriminating power of the obtained features. Contrast stretching, adaptive histogram equalization, and histogram equalization are methods for improving contrast [30]. In this study, we applied adaptive histogram equalization. This image preprocessing technique was applied to enhance contrast in images. It calculates various histograms, each related to a distinct portion of the image, and uses them to reallocate the luminance values of the image [30].

## Results

In this study, the performance of the newly proposed MobileNetV1 backbone for the Mask R-CNN architecture was compared to that of existing ResNet backbones. We used the mAP and AUC measures to evaluate how well each backbone performed on segmented images of the nasal septum. The mAP scores for the models with various backbones are shown in Table 1.

**Table 1:** Performance of the models based on mean average precision (mAP).

Models	Contrast enhancement mAP	mAP of nonprocessed images
ResNet50	0.911	0.731
MobileNetV1	0.866	0.813
ResNet101	0.843	0.752

With a mAP of 0.911, the Mask R-CNN with CEH-ResNet101 demonstrated its accuracy in detecting and segmenting nasal septum images. The Mask R-CNN with CEH-ResNet50 achieved a mAP of 0.866, indicating its high performance and effectiveness. With a mAP of 0.843, our proposed Mask R-CNN with CEH-MobileNetV1 also produced encouraging results. Comparatively, the mAP for the Mask R-CNN with ResNet50 was 0.731, and the mAP for the Mask R-CNN with MobileNetV1 (without CEH) was 0.813. The mAP for the Mask R-CNN with ResNet101 was 0.752.

As shown in Table 2, the Mask R-CNN with CEH-ResNet101 once more outperformed all other models, with an AUC of 0.921. An AUC of 0.864 demonstrates the Mask R-CNN’s good performance with CEH-ResNet50. The Mask R-CNN with CEH-MobileNetV1, which was recently incorporated, demonstrated competitive outcomes with an AUC of 0.843. The AUC for the Mask R-CNN with ResNet50 was 0.732, while the AUC for the Mask R-CNN with MobileNetV1 (without CEH) was 0.814. Finally, an AUC of 0.831 was obtained using the Mask R-CNN with ResNet101.

**Table 2:** Performance of the models based on the area under the curve (AUC).

Models	Contrast enhancement AUC	No processing AUC
ResNet50	0.921	0.732
MobileNetV1	0.864	0.814
ResNet101	0.843	0.831

## Discussion

### Principal Findings

In recent years, there has been an increase in the number of research on the application of AI in image analysis [31]. AI algorithms have been used for the detection and localization/ segmentation of anatomical areas in 3D image sources, such as computed tomography and CBCT [32,33]. CBCT has proven to be an efficient low-radiation dose modality for nasal septum and midfacial structures [34]**.** To the best of our knowledge, there are no studies highlighting the role of AI in the detection of NSD. In our study, we used Mask R-CNN for the detection of NSD in cropped coronal CBCT scans.

Mask R-CNN was not designed with pixel-to-pixel network input and output synchronization in view. RoI Pooling, which performs coarse spatial quantization for feature extraction, currently the core mechanism for managing instances, serves as an example of this approach. The network structure follows the design outlined by Bienias et al [35].

The multidimensional feature extraction and information fusion processes are carried out by the feature pyramid network and region proposal network of the backbone network, while the region proposal network additionally generates and offers target candidate regions based on extracted feature maps and classifications. Target instance segmentation is completed once the RoIAlign is used to correct the target region and integrate it with a faster R-CNN [36]. A simple, quantization-free layer called RoIAlign precisely records exact spatial locations. It improves mask accuracy by 10% to 50% when more severe localization procedures are used. By separating class prediction from mask prediction and relying on the network’s RoI classification branch, we can identify the category. Using data from previous ablation experiments, Mask R-CNN outperforms all contemporary single-model solutions to the COCO instance segmentation task [21].

In our study, Mask R-CNN with CEH-ResNet101 and CEH-ResNet50 proved to be the best performers, demonstrating high mAP and AUC values. However, the Mask R-CNN with CEH-MobileNetV1 technique, while not the top performer, delivered encouraging outcomes and showcased its potential as a portable backbone for nasal septum image detection and segmentation.

The MobileNetV1 backbone has been introduced, expanding the pool of options available to academics and professionals tasked with nasal septum image segmentation studies. We have demonstrated that the new backbone may provide an outstanding trade-off between accuracy and computational cost by comparing its performance to the well-established ResNet backbones. The MobileNetV1 backbone can now be considered by researchers as a workable substitute in situations where computing resources are constrained or real-time performance is essential.

This study offers an important new understanding of the effectiveness of the Mask R-CNN architecture with different backbones for detecting and segmenting images of the nasal septum. Professionals looking for effective but precise solutions now have a vital choice with the development of CEH-MobileNetV1 as a new backbone. These findings, we believe, will pave the way for more developments in medical image segmentation and encourage more study in this area.

### Limitations

One of the limitations of our study was the utilization of cropped 2D CBCT images for the detection of NSD. Future studies can be carried out using 3D CBCT images and AI software for NSD detection [29,37]. The limited number of images used for training and validation is another limitation of this study, which was due to the limited availability of CBCT images with large FOV. Most of the dental CBCT images are carried out on small and medium FOV, which means there were only a very limited number of large FOV scans that could be used for the study. Furthermore, the data used in our study were unbalanced (138 images with a deviated septum against 66 images with a nondeviated septum). To partially overcome this problem, we augmented the training data to increase the data to 5 times the original number of the deviated classes. The other limitation of the study is that only one dimension of the nasal septum was visualized. NSDs can be visualized in both the vertical (coronal) and horizontal (axial) planes [38]. Future studies can be conducted using AI-based detection of multiplanar views of the nasal septum.

### Conclusions

Nasal deviation has been examined, and it is crucial to create a model that can detect potential nasal deviation in real time. Mask R-CNN was used in this study to detect a deviated nasal septum, along with the different backbones Resnet101, ResNet50, and MobileNetV1. CEH was also applied to the images to improve the model’s performance, and it was found that CEH-ResNet101 outperformed all the other models used in the study, achieving a 0.911 mAP. This technique also demonstrates that CEH can enhance model performance when it comes to the detection of deviated nasal septum. The 3 distinct backbones used in this study—ResNet101, ResNet50, and MobileNetV1—show an acceptable degree of performance so far in detecting and segmenting deviated nasal septa. However, the performance of the models may be improved by using more datasets. To increase performance, we will use new backbones and enhanced backbone topologies in the future.

## Abbreviations

AI: artificial intelligence
AUC: area under the curve
CBCT: cone beam computed tomography
CEH: contrast enhancement
FOV: field of view
mAP: mean average precision
NSD: nasal septal deviation
R-CNN: region-based convolutional neural networks
RoI: Region of Interest
RoIAlign: Region of Interest alignment technique

## Data Availability

The data used in this study can be accessed at figshare [39].
